# Therapeutic peptides for coronary artery diseases: in silico methods and current perspectives

**DOI:** 10.1007/s00726-024-03397-3

**Published:** 2024-05-31

**Authors:** Ayca Aslan, Selcen Ari Yuka

**Affiliations:** 1 Department of Bioengineering, Faculty of Chemical and Metallurgical Engineering, Yildiz Technical University, Esenler, Istanbul, Turkey; 2 Health Biotechnology Joint Research and Application Center of Excellence, Esenler, Istanbul, Turkey

**Keywords:** Coronary artery disease, Atherosclerosis, Peptide therapeutics, In silico modeling, Molecular docking, Molecular dynamics simulations

## Abstract

Many drug formulations containing small active molecules are used for the treatment of coronary artery disease, which affects a significant part of the world’s population. However, the inadequate profile of these molecules in terms of therapeutic efficacy has led to the therapeutic use of protein and peptide-based biomolecules with superior properties, such as target-specific affinity and low immunogenicity, in critical diseases. Protein‒protein interactions, as a consequence of advances in molecular techniques with strategies involving the combined use of in silico methods, have enabled the design of therapeutic peptides to reach an advanced dimension. In particular, with the advantages provided by protein/peptide structural modeling, molecular docking for the study of their interactions, molecular dynamics simulations for their interactions under physiological conditions and machine learning techniques that can work in combination with all these, significant progress has been made in approaches to developing therapeutic peptides that can modulate the development and progression of coronary artery diseases. In this scope, this review discusses in silico methods for the development of peptide therapeutics for the treatment of coronary artery disease and strategies for identifying the molecular mechanisms that can be modulated by these designs and provides a comprehensive perspective for future studies.

## Introduction

Approximately 20.5 million people died in 2021 due to cardiovascular diseases, the leading cause of mortality worldwide. Approximately 9.44 million of these deaths were due to coronary artery disease (CAD) (Lindstrom et al. 2022; Vaduganathan et al. 2022).

The most common cause of CAD is atherosclerosis (Hansson 2005). Atherosclerosis is an inflammatory disease characterized by plaque formation in the intima of arteries due to cholesterol. Current treatment modalities for atherosclerosis include lifestyle modifications (food, sports, stress, smoking, etc.) and the use of cholesterol-lowering drugs (statins, etc.) or drugs that prevent the formation of plaque and clotting in the vessel (aspirin, clopidogrel, ticlopidine, etc.). On the other hand, the growth of atherosclerotic plaque or the rupture of plaque leads to vascular occlusion, resulting in myocardial infarction (MI), the most important clinical sign of CAD (Malek et al. 1999). Following occlusion of the vascular for 15–30 min, necrosis begins to develop in the heart muscle, and the infarct area expands over time. (Hermens et al. 1992). Therefore, the most important stage in the treatment of MI is the rapid restoration of blood flow to the infarct-related vascular without allowing myocardial necrosis and its consequences to occur.

For this purpose, cardiac reperfusion procedures such as percutaneous coronary intervention (PCI) and/or thrombolytic therapy (reteplase, tenecteplase, etc.) are administered in the early stage to open the occluded artery and provide reperfusion (Members et al. 2012; McKelvie et al. 2013). Coronary artery bypass grafting (CABG) is performed for coronary occlusions that cannot be opened with PCI (Thielmann et al. 2006). The treatment approach to prevent ventricular remodeling, which initially appears to be a compensatory mechanism but leads to ventricular dysfunction over time, involves the use of antiremodeling drugs (angiotensin-converting enzyme (ACE) inhibitors, β adrenergic blockers, etc.) to suppress neurohumoral activation, which plays a primary role in remodeling (Pitt et al. 2006).

Drug formulations derived from natural sources or chemically synthesized compounds, which are characterized as small molecules used today for CAD, constitute a large part of the pharmaceutical market today due to their low production cost, ease of oral intake and good membrane permeation profile (Imai and Takaoka 2006). In addition to their advantages, these small molecule formulations are inadequate for inhibiting protein–protein interactions that occur over extensive surface areas and have low specificity (Wang et al. 2022). One of the favorable options to overcome these issues associated with small molecules has been the development of therapeutic proteins or antibodies. Peptides in particular offer the advantages of lower immunogenicity and lower cost of production than other biological therapeutics such as proteins and antibodies, while having a similar target-specific affinity profile (Muttenthaler et al. 2021). In addition, peptides can also be designed with properties that allow them to pass through the cell membrane compared to these large biological molecules (Xie et al. 2020). Despite these superior properties, the most prominent issue for the clinical application of peptides is that they exhibit poor drug-like properties (absorption, low stability to proteolytic digestion, and fast clearance) (Petri et al. 2022). Peptidomimetics are chemical compounds capable of interacting with biological targets while retaining the ability to elicit the same biological response by emulating the structural and functional characteristics of natural peptides (Gatto et al. 2021).

Approaches to the application of peptides and mimetics developed to date for these promising approaches in the treatment of CAD have been discussed previously (Litmanovich et al. 2022a, b; Recio et al. 2017). In this review, we discuss the mechanisms that need to be addressed in the development of therapeutic peptides and peptide-derived molecules for CAD, the current and potential targets that can act on these mechanisms, and the effective strategies that can be developed, especially those based on structural biology.

## Brief overview of the pathogenesis of coronary diseases

**Fig. 1:**
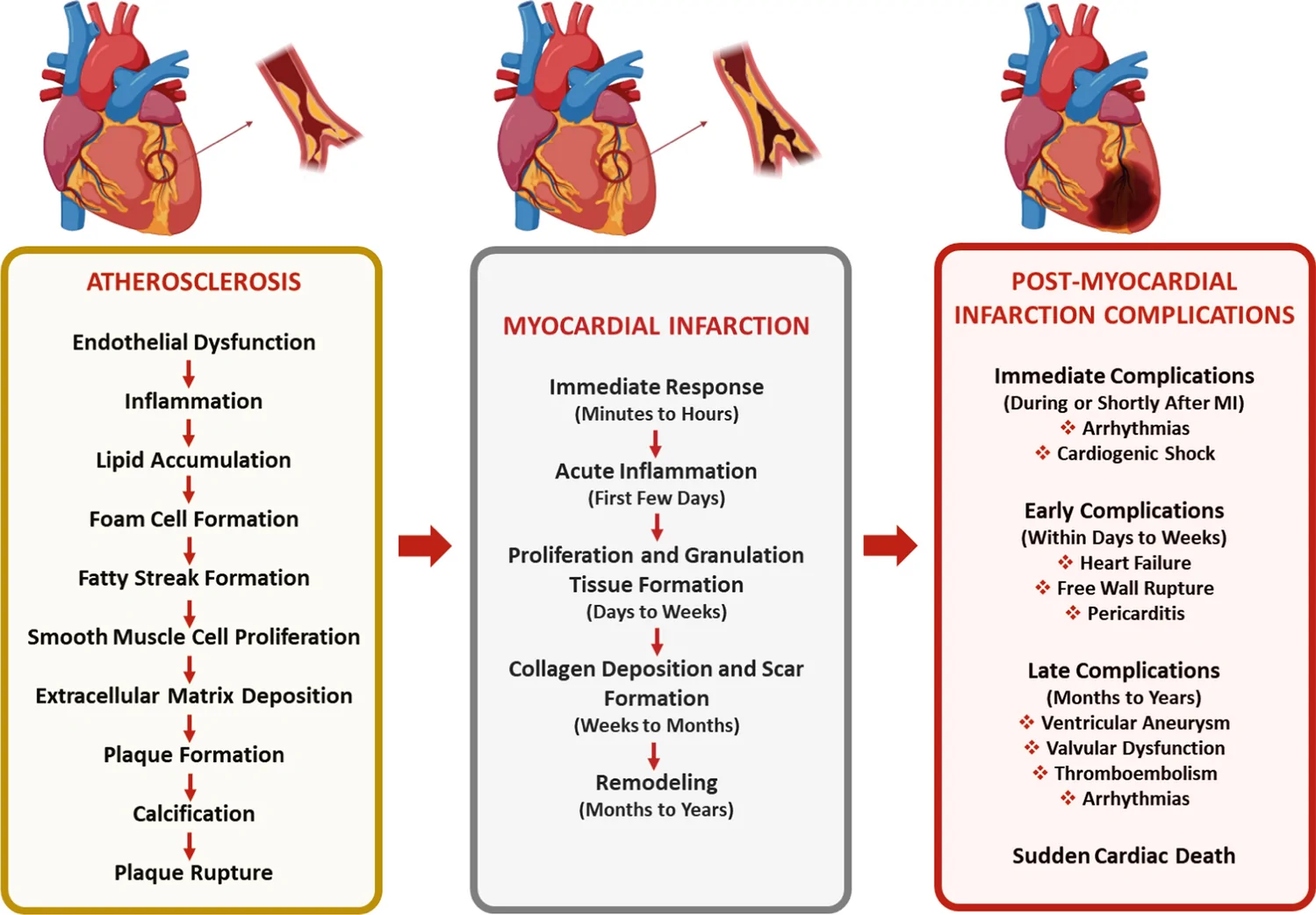
Summary of the pathophysiology from atherosclerosis to post-myocardial infarction

Atherosclerosis is an inflammatory disease that develops in the layer of arteries and is characterized by endothelial dysfunction, low-density lipoprotein (LDL) oxidation, inflammation, and plaque formation (Fig. 1) (Hansson 2005; Heitzer et al. 2001).

The endothelium has an important role in controlling the transportation of nutrients and the elimination of waste, as well as in managing processes such as inflammation, blood clot formation, and coagulation. It accomplishes this by releasing primary autocrine and paracrine mediators, such as nitric oxide (NO), prostaglandins, hyperpolarizing factors, endothelin, and angiotensin II (Beckman et al. 2004). These mediators maintain balance by regulating both the widening and narrowing of blood vessels, thrombosis and anticoagulation, and the adjustment of inflammatory responses. Among these mediators, NO is the best characterized. NO is a very potent vasodilator synthesized by nitric oxide synthase and is considered an atheroprotective molecule because it prevents atherogenesis and its complications (Aggio et al. 2013; Chen et al. 2018). Nevertheless, factors such as smoking, diabetes mellitus, or elevated superoxide levels promote the synthesis of pro-atherogenic cytokines, adhesion molecules, and chemokines by triggering NFκB activation via heat shock proteins (Förstermann et al. 2017; Gimbrone and García-Cardeña 2016). These mediators inhibit NO synthesis. Thus, the endothelium loses both its selective permeability and antithrombic properties.

As a result of damage to the endothelium, atherogenic LDLs, whose number increases in the blood, pass to the subendothelial layer where they can be modified. LDL passing into the subendothelial layer is recognized by macrophages, but the rate of phagocytosis of LDLs by macrophages is initially low because there are few LDL receptors (LDLRs) on macrophages (Leiva et al. 2015).

For LDL to be better recognized by macrophages and consequently foam cells to form, LDL undergoes two-stage oxidative modifications by endothelial cells, smooth muscle cells in the intima and media layer and macrophages (Leiva et al. 2015; Parthasarathy et al. 2010). In the initial phase, minimally modified LDLs (mm-LDL), LDLs in which the ApoB-100 structure is unchanged, are acquired. However, since these mm-LDLs are still recognized by LDLR, foam cell formation is not observed. These LDLs act as chemoattractants for monocytes, increasing their population. In the second stage, monocytes are recruited to the lesion, where they transform into macrophages and contribute to their oxidative capacity. In the second stage modification, LDL oxidation is completed as a result of a change in the structure of ApoB-100, and oxidized LDLs are recognized by scavenger receptors on macrophages (Chistiakov et al. 2016; Greaves and Gordon 2009). Accordingly, the rate of phagocytosis of LDLs increases. In addition, as a result of macrophage uptake of LDL by scavenger receptors, there is no regulation of cholesterol content, as in LDLR recognition; consequently, cholesterol accumulation occurs. Cholesterols begin to form fat droplets inside the cell, and the macrophage is transformed into a lipid-laden foam cell. Foam cells are the precursor cells of atherosclerosis (Hermens et al. 1992). However, lipid uptake by macrophages activates the NF-kB pathway, generating an inflammatory response, and cytokines such as tumor necrosis factor (TNF) and interleukin-1 (IL-1) are produced to increase monocyte recruitment into the environment (Peled and Fisher 2014; Kim et al. 2011; Takahashi et al. 2021). These cytokines act on the endothelium of capillaries at the site of infection. As a result of cytokine-endothelial cell interactions, the expression of two adhesion molecules, E-selectin and P-selectin, is stimulated. Carbohydrate molecules on the surface of circulating monocytes bind weakly to these selectins. Although monocytes initially adhere to the endothelium, blood flow dissolves this weak binding. Subsequently, the blood flow begins to slow, and the viscosity of the blood increases as a result of the increased permeability of the vessels. This increases the migration of monocytes to the vascular endothelium. The increased migration of monocytes and their differentiation into macrophages leads to an increase in the number of foam cells and consequently to the growth of atherosclerotic lesions and plaque formation (Bobryshev 2006). As a result of atherosclerotic plaque rupture, thrombus formation occluding the vascular lumen is observed, resulting in MI, which is the most crucial clinical sign of CAD (Fig. 1) (Malek et al. 1999).

MI can be defined as cardiac necrosis resulting from prolonged ischemia (Antman et al. 2000). If the occluded artery during MI is not opened within approximately 20 min, apoptosis is observed in approximately 500 cardiomyocytes (CMs) per second in the area where blood flow cannot be provided, and myocardial necrosis begins to occur over time (Hermens et al. 1992). This leads to impaired contractility of the myocardium, and the heart enters the ventricular remodeling phase, which is part of the compensatory mechanism (Azevedo et al. 2015; Sutton and Sharpe 2000; Burchfield et al. 2013). While post-infarction remodeling affects globally, there are distinct differences in the pathophysiological responses within the infarcted and noninfarcted domains. In the infarcted area, remodeling involves processes such as myocardial cell death, infiltration of inflammatory cells, activation of matrix metalloproteinases (MMPs), and degradation of the extracellular matrix. All of these factors contribute to the enlargement of the infarcted area and thinning of the heart wall. Within a few days following infarct enlargement, transforming growth factor (TGF)-β1 secretion, myofibroblast transformation, tissue angiotensinogen II and aldosterone production, and collagen synthesis and storage occur thus scar tissue formation begins (Burchfield et al. 2013; Prabhu 2005; Zornoff et al. 2009). In contrast to the infarct area, in the noninfarct area, hypertrophy occurs to compensate for the loss of the contractile area. This pathophysiological adaptation occurs as a result of neurohormonal pathways such as mechanical load, increased adrenergic, autocrine and paracrine mediators, and oxidative stress. This chain of events, which initially starts as compensatory, results in pathological hypertrophy, contractile dysfunction, CM loss, and fibrosis, leading to enlargement of the ventricle, development of spherical geometry, systolic and diastolic dysfunction, and decreased ejection fraction in the long term (Prabhu 2005; Gajarsa and Kloner 2011). All of these consequences can lead to complications such as thrombus formation, and heart failure and may cause the patient to die or reduce the standard of living (Fig. 1) (Grasso and Brener 2014).

## In silico methods for peptide design

Therapeutic peptides are a considerable subgroup of pharmaceuticals and are promising agents that can exhibit unique properties based on amino acid (aa) sequence and content. As a result of significant advances in structural biology, genetic engineering and recombinant technologies, the development of peptide-based agents has accelerated in the twenty-first century. In addition to their effects on metabolic, cardiovascular, respiratory and urological diseases, these peptide therapeutics can also be used as antimicrobial agents (Fisher et al. 2019; Sloan 2019; Peterson and Barry 2018). Therapeutic peptides, which have great potential due to their unique nature, require multidisciplinary approaches to be used together in the design of therapeutic peptides. In peptide design, it is possible to optimize the half-life, biological activity and water solubility of peptides under physiological conditions, and to perform appropriate modifications accordingly (Goodwin et al. 2012; Henninot et al. 2018).

**Fig. 2:**
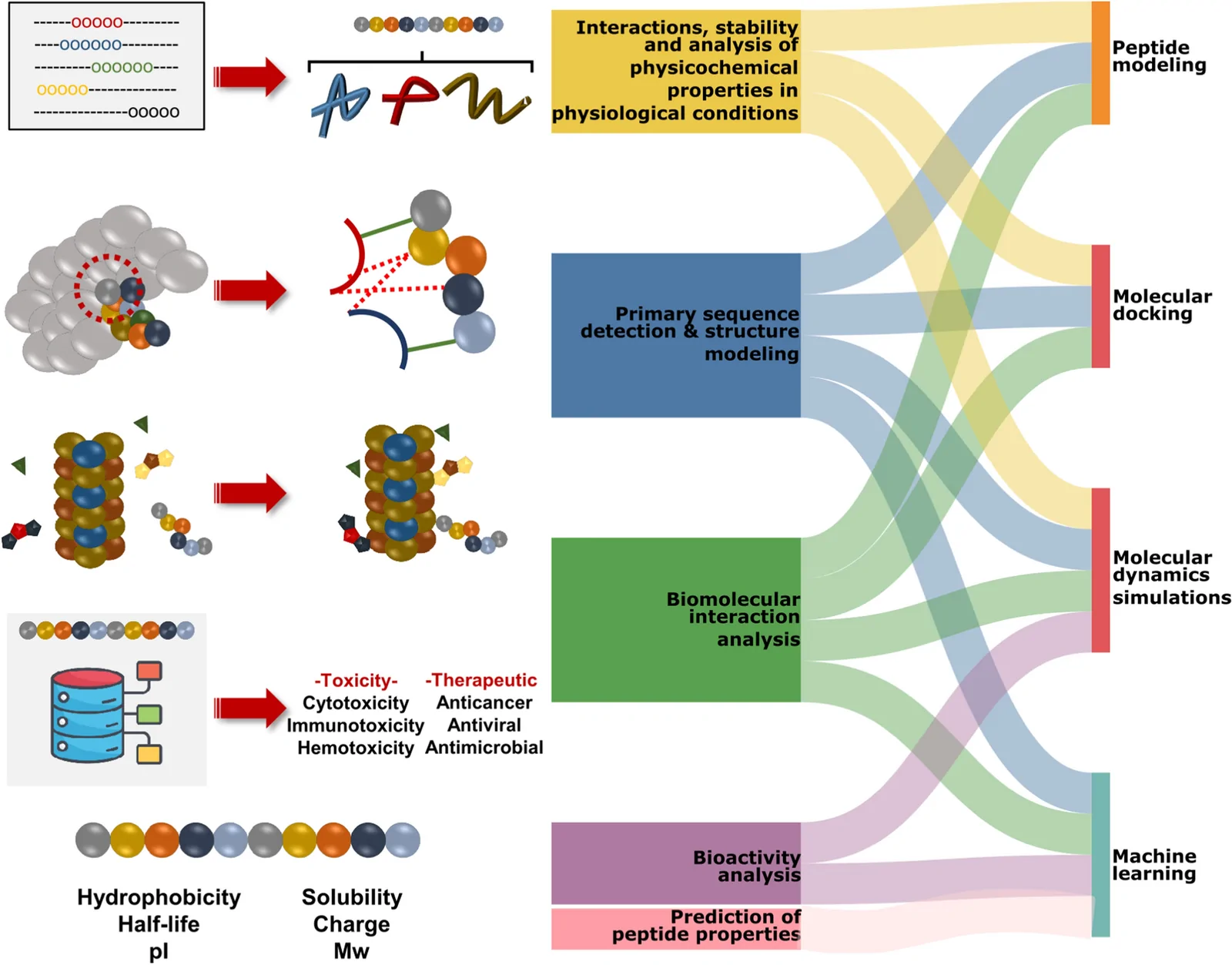
Summary of in silico strategies for peptide design

Therapeutic peptide design methods based on structural biology encompass a multifaceted framework that combines biological, chemical, and physicochemical analyses. First, aa sequences, residues and hotspot aa’s are derived from interacting biomolecules using data obtained from known crystal structure databases of the target protein to be used for therapeutic activity. These sequences are modeled to determine their conformational properties under physiological conditions, their interactions with the target protein are explored by in silico methods using the model, and the properties of the sequence under physiological conditions are analyzed by computational methods (Fosgerau and Hoffmann 2015; Gupta et al. 2023). In these analyses, properties such as the stability, charge, polarity or water solubility of peptides according to their aa content are evaluated to determine the most proper peptide structure for the intended use. In addition, molecular docking, dynamics simulations or machine learning-based biological activity prediction tools can be used to reduce the need for experimental procedures by providing insights into the activity and interactions of peptides (Table 1) (Fig. 2).

**Table 1:** Methodologies and the most common tools used in peptide design

Methodology	Name	Tool/Server	Ref
Peptide Feature	ProtParam	https://web.expasy.org/protparam/	Gasteiger et al. (2005)
	Pep-Calc	https://pepcalc.com/	Lear and Cobb (2016)
	PDAUG	Galaxy based toolset	Joshi and Blankenberg (2022)
Peptide structure prediction	PEP-FOLD(3–4)	https://bioserv.rpbs.univ-paris-diderot.fr/services/PEP-FOLD3/ https://bioserv.rpbs.univ-paris-diderot.fr/services/PEP-FOLD4/	Lamiable et al. (2016; Rey et al. (2023)
	PEPstrMOD	http://osddlinux.osdd.net/raghava/pepstrmod/	Singh et al. (2015)
	LassoHTP	Standalone	Juarez et al. (2023)
	APPTEST	https://research.timmons.eu/apptest	Timmons and Hewage (2021a)
Docking (Template-based)	GalaxyPepDock	https://galaxy.seoklab.org/	Lee et al. (2015)
Docking (Local docking)	AutoDock Vina	Standalone	Eberhardt et al. (2021)
	Rosetta FlexPepDock	Standalone and http://flexpepdock.furmanlab.cs.huji.ac.il/	Raveh et al. (2011)
	DynaDock	Not public	Antes (2010)
	HADDOCK	https://milou.science.uu.nl/services/HADDOCK2.2	Dominguez et al. (2003)
	DINC 2.0	https://dinc.kavrakilab.org/	Antunes et al. (2017)
	Gold	Standalone	Jones et al. (1997)
	Surflex-Dock	Standalone	Jain (2007)
	ADCP	Standalone	Zhang and Sanner (2019)
***	HPEPDOCK	http://huanglab.phys.hust.edu.cn/hpepdock/	Zhou et al. (2018)
Docking (Global docking)	MDockPeP	https://zougrouptoolkit.missouri.edu/mdockpep/	Xu et al. (2018)
	ClusPro PeptiDock	https://peptidock.cluspro.org/	Porter et al. (2017)
	pepATTRACT	https://bioserv.rpbs.univ-paris-diderot.fr/services/pepATTRACT/	Vries et al. (2017)
	ZDOCK	https://zdock.umassmed.edu/	Pierce et al. (2014)
Molecular dynamics simulations	AMBER	https://ambermd.org/	Salomon-Ferrer et al. (2013)
	GROMACS	https://www.gromacs.org/	Abraham et al. (2015)
	CHARMM	https://www.charmm.org/	Brooks et al. (2009)
	NAMD	https://www.ks.uiuc.edu/Research/namd/	Phillips et al. (2005)

*** specifies the tool that is capable of both local and global docking

### Peptide structure modeling

To analyze peptide properties or interactions in silico, several tools can directly receive the peptide primary sequence and model it in the pipeline, while other tools require the peptide sequences to be modeled first. Obtaining peptide models with high accuracy ensures accurate results in subsequent analyses. Today, there are various easy-to-use tools that are easily accessible. For example, PEP-FOLD(3–4) assembles fragments from the aa sequence using a structural alphabet derived from the Hidden Markov Model (HMM), enabling peptide modeling in the 5–50 aa range (Lamiable et al. 2016). Another tool, PEPstrMOD, models 7–25 aa-long peptide sequences consisting of natural, non-natural or modified aa residues by incorporating modified residues using force field libraries (Singh et al. 2015). Lasso peptides produced with posttranslational modifications, with superior thermal stability, protease resistance, and antimicrobial activity, can be modeled with the LassoHTP tool, by generating a structure and conformational ensemble (Juarez et al. 2023). However, one of the most advanced approaches for peptide modeling is AlphaFold (Varadi et al. 2022; Jumper et al. 2021). It, which enables the prediction the structure of proteins and peptide sequences by using deep learning and neural network models, is one of the most cutting-edge approaches to use for novel peptide design for targeting specific proteins. The accuracy of peptide structure modeling algorithms typically varies depending on peptide length or secondary structures (McDonald et al. 2023). Therefore, instead of using a precise modeling algorithm in peptide design studies, a comprehensive and comparative evaluation of the outputs of several modeling approaches may yield more accurate designs. On the other hand, modeling of peptide structures does not assure high therapeutic efficacy against CAD does, but it is evident that these methods are critical for generating in silico interaction models with higher accuracy in further analysis of the interactions of the designed peptides with targeted proteins in target CAD molecular processes.

### Molecular docking

In order to make peptide designs more accurate, methods such as machine learning, molecular docking or molecular dynamics simulations have started to be used along with conventional peptide design methods. Molecular docking techniques that can be used in peptide design can be categorized into three main classes (Ciemny et al. 2018). The first is the template (or homology) based docking technique, which uses known complexes as templates to infer the interactions of input peptides and proteins (Szilagyi and Zhang 2014). The most well-known template-based molecular docking methods are GalaxyPepDock (Ciemny et al. 2018; Weng et al. 2020). The second is the global (i.e., blind) docking technique, which docks the peptide sequence provided in the input file by rendering all possible binding coordinates without specifying a specific binding site on the target protein. The most used global molecular docking methods include the pepATTRACT, MDockPeP, CABS-Dock, ClusPro PeptiDock, AnchorDock, PIPER-FlexPepDock and HPEPDOCK tools (Ciemny et al. 2018; Weng et al. 2020). The integration of template-based methods often improves the accuracy of global molecular docking approaches (Johansson-Åkhe et al. 2020). The final docking strategy is the local analysis technique, which evaluates all possible binding within a box located at user-defined coordinates on the target protein. In these techniques, peptides can be provided in folded form or as a sequence. The most widely used local tools for peptide-protein molecular docking are AutoDock Vina (Vina) (Jokar et al. 2020), GOLD (Mahdavi and Moreau 2016), Surflex-Dock (Surflex), DynaDock, HADDOCK, PepCrawler, DINC, AutoDock CrankPep (ADCP), Rosetta FlexPepDock and HPEPDOCK (Ciemny et al. 2018; Weng et al. 2020). Virtual screening approaches utilizing different molecular docking tools have been introduced for CAD. When the appropriate docking method is adopted, these techniques provide unique tools for rapid and comprehensive evaluation of peptide libraries pooled from diverse sources. The most typical example of this is the docking-based virtual screening methods applied to develop inhibitory peptides against ACE, a hypertension mediator, from different natural sources (Wu et al. 2014). Thus, docking and screening of 54 tetrapeptides obtained from Salmo salar collagen by in silico proteolysis revealed the potential of PGAR and IGPR sequences (Yu et al. 2018). The selection of methods that use distinct model generation algorithms and scoring methods to study peptide-protein interactions is critical for the tool's performance and the current design’s accuracy. Benchmarking peptide-protein molecular docking tools shows that model input requirements (such as binding site size), which may vary depending on the docking tool, and the length of the peptide are crucial in the performance and accuracy of these approaches (Weng et al. 2020). Therefore, peptide properties and specific requirements must be well-defined in virtual screening approaches for peptides against proteins critical in CAD.

### Molecular dynamics simulations

Molecular dynamics simulations methods, which enable the simulation of all atoms of biomolecules, provide advanced methods for the comprehensive analysis of peptide interactions and stability. Force fields such as AMBER (Ponder and Case 2003), CHARMM (Vanommeslaeghe et al. 2010), GROMOS (Schmid et al. 2011), and OPLS (Jorgensen et al. 1996) which can be used in MD simulations, affect the efficacy of MD results by varying the calculation of peptide and condition specific forces (Jephthah et al. 2021; Man et al. 2019; Conde et al. 2022). Molecular dynamics simulations are critical for demonstrating the experimental potential of peptides developed for therapeutic purposes, as simulation packages such as AMBER (Salomon-Ferrer et al. 2013), GROMACS (Abraham et al. 2015), CHARMM (Brooks et al. 2009) and NAMD (Phillips et al. 2005) can be used to comprehensively analyze interactions and stability. Notably, the algorithmic advancement and performance of MD simulations vary but are not the primary concern of this paper and have been addressed by numerous studies in the literature (Hospital et al. 2015). Natriuretic peptide receptor (NPR), which regulates many functions, such as blood pressure, cardiac hypertrophy, and fibrosis with various peptide interactions, is one of the biomolecules widely subjected to MD simulation studies. Although there are already many studies on the application of peptides involved in NPR interactions (atrial natriuretic peptide (ANP), B-type natriuretic peptide (BNP)), studies are in progress to develop stable peptide approaches that can achieve higher bioavailability. (Potter et al. 2009). For example, MD simulations have shown that Lebetin 2 (L2), a natriuretic-like peptide, exhibits more favorable interactions than does the natriuretic peptide BNP (Allaoui et al. 2022). Current studies aim to provide advanced solutions for therapeutic approaches from a multidisciplinary perspective. The self-assembly mechanisms, mechanical properties, and interactions with integrin and Natriuretic peptide receptor-C (NPR-C), which are highly expressed in cardiac tissue cells, have been extensively investigated by MD simulations of a complex composite containing VEGF-derived peptide (NYLTHRQ) sequences for cardiac tissue engineering applications (Mitchell et al. 2023). The scaffold produced as a result of their studies was reported to interact with both integrins and NPR-C but especially with higher affinity and stability with NPR-C receptor. MD simulations are also used for docking and collection of virtual screen libraries. For example, for the assembly of peptide sequences from natural sources, it is also useful to comprehensively analyze the conformational changes of the subject natural proteins and then dock peptides derived from the regions of critical residues (Tahir et al. 2020). The MD simulations clearly show the potential of in silico methods in therapeutic applications for cardiac tissue, both in understanding the underlying mechanisms in detail and in developing manipulable and state-of-the-art approaches. Although these state-of-the-art methods provide comprehensive analyzes for a deep understanding of peptide protein interactions, they also require a high-performance computing environment and time, especially due to the flexible nature of peptides (Yin et al. 2024). Therefore, current perspectives suggest that conventional computational models and simulations can be improved through machine learning methods.

### Machine learning techniques

Several tools and servers with machine learning methods hold great promise for predicting the biological activity (antimicrobial, antiviral, etc.) of peptides (Ali et al. 2023). These approaches, such as ENNAVIA (Timmons and Hewage 2021b), CAMPR4 (Gawde et al. 2023), DeepACP, (Chen et al. 2021), iAMPCN (Xu et al. 2023), and sAMPpred-GAT (Yan et al. 2023), which were developed to address specific biological activities, such as antimicrobial or anticancer activities, are based on the evaluation of input sequences by utilizing peptide libraries containing experimentally validated peptide sequences, and applying various methods, such as Random Forest (RF), Support Vector Machinesupport vector machine (SVM) or different types of Neural Network (NN) models. These approaches, which can be used to generate predictions, especially in antimicrobial peptide designs, due to their extensive library content, respond according to the properties evaluated on the basis of existing antimicrobial peptide sequences, depending on the aa content of the peptides. However, these methods lack detailed information on the biological activity of the sequences, cannot be evaluated by learning methods, and are limited by false positives due to the wide range of peptide properties. These methods, where parameters such as charge or polarity are considered in the framework, focus on evaluating the properties of the overall peptide sequence rather than target-specific interactions. Therefore, machine learning methods for assessing the bioavailability and undesirable toxicity (cytotoxicity, immunotoxicity, and hemotoxicity) of peptides are limited. Approaches are being developed to overcome these limitations. Plisson et al., applied several machine learning algorithms to hemolytic peptide and antimicrobial peptide datasets and emphasized that gradient boosting and extreme gradient boosting classifiers performed the best and even proposed 34 high-confidence nonhemolytic natural AMPs (Plisson et al. 2020). A more recent approach has shown that by enhancing the effectiveness of machine learning methods with molecular docking and dynamics simulations, it is possible to develop fine-tuned peptide designs that can achieve the desired biological activity (Zhang et al. 2023). In particular, the use of machine/deep learning methods, which are frequently used in the design of anticancer and microbial peptides and in the study of protein‒peptide interactions, in the design of novel therapeutic molecules for CAD could lead to significant advances (Varadi et al. 2022; Jumper et al. 2021; Lei et al. 2021). Predicting the biological activity of peptide sequences in cardiovascular diseases by ML methods does not currently appear to be applicable. This is mainly due to the lack of comprehensive and sufficient datasets and the missing features that determine the therapeutic effects of peptides. Therefore, as with antimicrobial or anticancer biological activity prediction methods, comprehensive datasets are needed to develop ML-based methods for identifying sequences that may be effective against specific mechanisms involved in cardiovascular diseases. On the other hand, ML methods can pave new frontiers for the prediction of peptide structures that may interact with critical targets in cardiovascular diseases (Bertoline et al. 2023; Tsaban et al. 2022). Inspired by AlphaFold2, the state-of-the-art example of this, it may be pioneered to create a library of the most favorable sequences for the active sites of proteins critical in cardiovascular diseases by training the interface features of protein–protein interactions (Yang et al. 2023). All of these observations suggest that the computational tools in structural biology may advance into more sophisticated technological platforms through the integration of machine learning techniques. However, concurrently, there is a need for much more comprehensive peptide libraries compiled with their respective features to accurately predict the biological activities of peptides on CAD.

## Current strategies of therapeutic peptides in CAD

In CAD, conventional small molecule formulations are generally used clinically due to their low manufacturing cost, ease of oral administration and good membrane permeability profile (Rossello et al. 2015). However, different reports show that the different therapeutics used for CAD have a wide side effect profile. For example, long-term beta-blocker therapy in stable CAD has been reported to have no significant effect on reducing ischemic events and to causeor causing a variety of side effects, including hypotension, bronchospasm, and peripheral vasoconstriction (Lee et al. 2022). In addition, chronic beta-blocker use has been associated with deterioration of lipid profiles and thus new-onset diabetes (Elliott and Meyer 2007). On the other hand, it has been reported that the use of acetylsalicylic acid (ASA), an antiplatelet drug, for 5 years may cause bleeding requiring treatment in 2–3 out of every 100 people (Collins et al. 2009). Statins, which are used to alleviate the consequences of lipoprotein cholesterol, are used to reduce the risk of cardiovascular death, recurrent MI, and stroke. Despite being one of the most cost-effective therapeutic interventions, this drug raises safety concerns due to geriatric-specific side effects and the potential to increase the incidence of diabetes (Rossello et al. 2015; Sattar et al. 2010).

All these findings have revealed the need for new perspectives instrategies for the treatment of CAD, and as a result, studies on high-potential peptide therapeutics have attracted increased amounts of attention. Its superior properties, such as target specificity, high potential for chemical and biological diversity, low accumulation in tissues and low toxicity, have highlighted peptides as potential candidates for the treatment of CAD, and clinical trials of CAD-targeted peptides have started, starting with peptides derived from known protein interactions. (Wang et al. 2022; Ichiki et al. 2019; Meems and Burnett 2016) (Table 2). On the other hand, depending on the aa composition of the peptides, their metabolic stability, bioavailability, half-life or immunological properties should be considered (Recio et al. 2017). At this point, the use of in silico approaches offers state-of-the-art methods to eliminate such undesirable properties of peptides. As mentioned, current studies are in the direction of achieving optimal therapeutic efficacy in peptide designs against CAD through in silico methods that allow many predictions to be made before wet laboratory applications. In this chapter, the most recent studies on therapeutic peptide applications against CAD are addressed.

**Table 2:** The therapeutic peptides for CAD currently being used or under phase trials

Peptide name	Aim of the study	Sequences	Phase	Clinical trials ID
FX06 (Fibrinogen beta chain)	To evaluate whether FX06, which prevents leukocyte migration through the gap junctions of endothelial cells, limits infarct size following acute MI	MKHLLLLLLCVFLVKSQGVNDNEEGFFS	Phase II	NCT00326976
Nesiritide (B-type natriuretic peptide)	To search the effect of BNP on endothelial dysfunction caused by coronary angioplasty	SPKMVQGSGCFGRKMDRISSSSGLGCKVLRRH	Phase IV	NCT00262574
Nesiritide (B-type natriuretic peptide)	To compare the hemodynamic and clinical effects of the study drug, Nesiritide to those of intravenous nitroglycerin or placebo, when added to the standard-of-care therapy that is usually administered in the treatment of patients with worsening congestive HF	SPKMVQGSGCFGRKMDRISSSSGLGCKVLRRH	Phase III	NCT00270374
Nesiritide (B-type natriuretic peptide)	To assess whether nesiritide when given with standard-of-care therapies, helps preserve kidney function in HF patients undergoing heart bypass graft surgery that requires the use of a cardiopulmonary bypass machine	SPKMVQGSGCFGRKMDRISSSSGLGCKVLRRH	Phase II Phase III	NCT00653042 NCT00530361
Nesiritide (B-type natriuretic peptide)	To determine whether human BNP has beneficial effects on the heart's pumping function and prevent adverse left ventricular remodeling post-MI	SPKMVQGSGCFGRKMDRISSSSGLGCKVLRRH	Phase I Phase II	NCT00252213 NCT00573144
Nesiritide (B-type natriuretic peptide)	To evaluate whether recombinant human BNP can reduce microcirculatory obstruction and reduce the area of MI in STEMI patients undergoing primary PCI	SPKMVQGSGCFGRKMDRISSSSGLGCKVLRRH	Not Applicable	NCT05723315
Liraglutide (GLP-1 analog)	To evaluate the effects of liraglutide on myocardial reperfusion in patients with acute STEMI	HAEGTFTSDVSSYLEGQAAKEEFIAWLVRGRG	Not Applicable	NCT02507128
Liraglutide (GLP-1 analog)	To search the changes in arterial stiffness, endothelial glycocalyx thickness and coronary reserve flow after metformin or GLP-1 receptor agonist treatment in patients with T2DM without CAD, and in patients with T2DM and CAD	HAEGTFTSDVSSYLEGQAAKEEFIAWLVRGRG	Not Applicable	NCT03010683
Exenatide (GLP-1 Analog)	To evulate the potential of GLP-1 to positively affect both cardiac function and glucose metabolism in cardiac surgery patients with coronary atherosclerosis with and without T2DM	HGEGTFTSDLSKQMEEEAVRLFIEWLKNGGPSSGAPPPS	Phase III	NCT01373216
GLP-1	To evaluate whether GLP-1 given during elective coronary angioplasty and stenting will reduce the increase in cardiac troponin (a measure of heart muscle damage) and protect the heart	HAEGTFTTSDVSYSSTLEGQAAKEFIAWLVKGR	Phase I Phase II	NCT02128022 NCT02127996
GLP-1	To search the effects of acute hyperglycemia and its modulation by GLP-1 on myocardial perfusion in T2DM	HAEGTFTTSDVSYSSTLEGQAAKEFIAWLVKGR	Not Applicable	NCT01021865
Apelin	To evaluate the effect of Apelin, which strengthens the heart pump, on heart contraction in people with HF	MNLRLCVQALLLLWLSLTAVCGGSLMPLPDGNGLEDGNVRHLVQPRGSRNGPGPWQGGRRKFRRQRPRLSHKGPMPF	Not Applicable	NCT01179061
ANP	To evaluate whether ANP as an adjunctive therapy for acute MI reduces myocardial infarct size and improves regional wall motion	SLRRSSCFGGRMD RIGAQSGLGCNSFRY	Not Applicable	NCT00212056

*ANP* Atrial natriuretic peptide, *BNP* Brain natriuretic peptide, *CAD* Coronary artery disease, *GLP-1* Glucagon-like peptide 1, *HF* Heart failure, *MI* Myocardial infarction, *T2DM* Type 2 diabetes mellitus, *PCI* Percutaneous Coronary Intervention, *STEMI* ST-Segment elevation myocardial infarction

### Structural proteins and ion channels

**Fig. 3:**
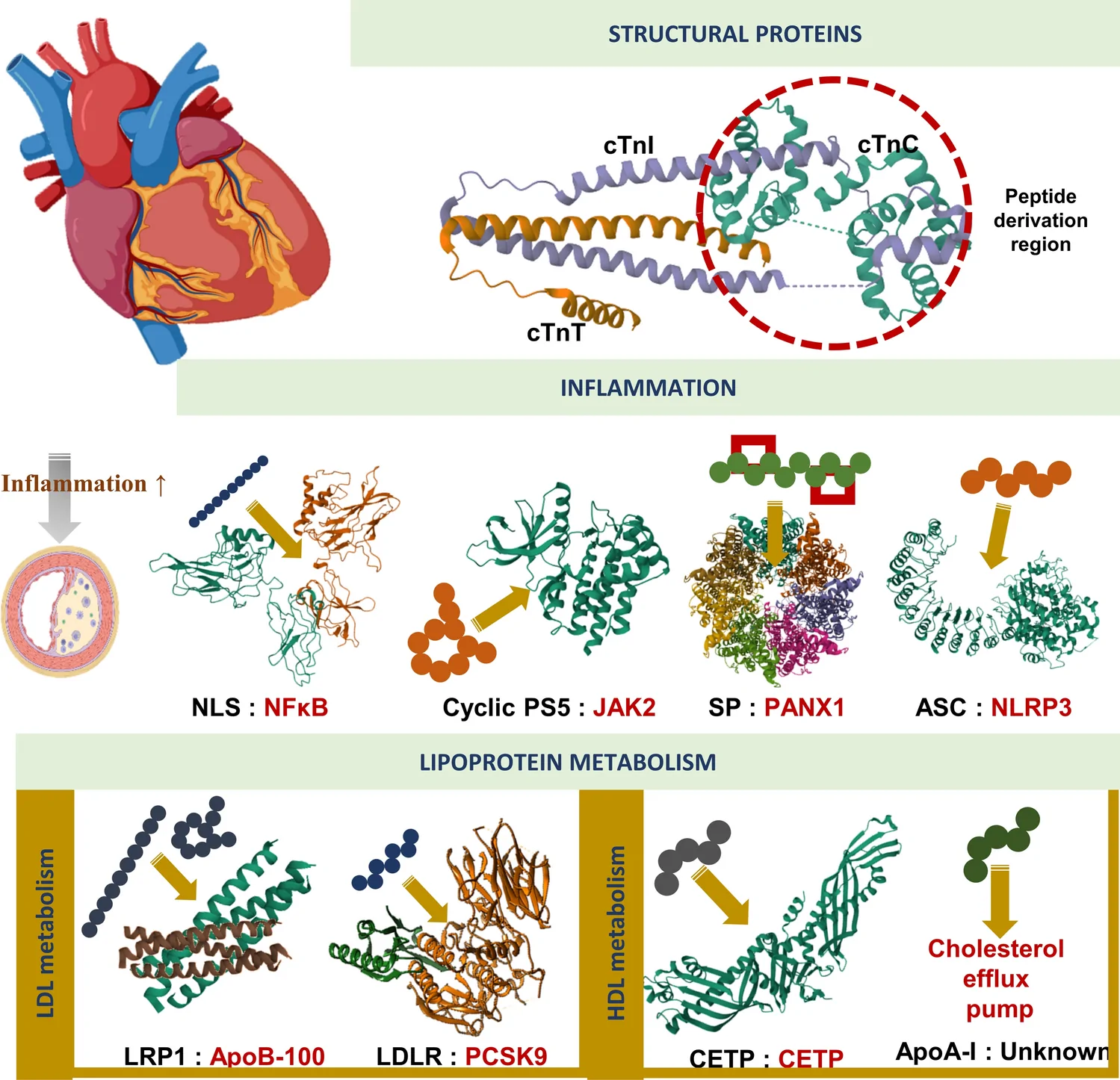
Current strategies of peptide-based CAD therapeutics. *NLS* peptide of NF-κB nuclear localization sequence, *PS5* peptide mimetic of SOCS1 protein, *SP* Stapled peptide of ^10^Panx1 Analog, *ASC* peptide derived from apoptosis-associated speck-like protein containing a CARD, *CR9* peptide derived from a particular domain of LDL, *LRP1* LRP1 peptide derived from different fragments, different cyclic designs and modifications, *ApoA-I* mimetic peptide derived from ApoA-I, *CETP* peptide derived from the C-terminal tail of the CETP protein (self binding mechanism). Due to the complex structure of the APO-B 100 protein, crystallography of the whole complex protein is not available. Therefore, APO-B 100 is shown here only as a demonstration. For the protein structures shown in Figure, the PDB database was used and UCSF Chimera (v1.16) was used for posing (Pettersen et al. 2004)

To restore the healthy function of cardiac tissue, several approaches are emerging that can interact with various structural proteins and thereby regulate ion channels. One of the clear examples of this phenomenon is the pioneering studies on cardiac troponin (Tn) by Genchev et al., to ensure a healthy cardiac contraction process, the intermolecular interactions and multistep contractile function of Tn must be well characterized (Fig. 3). MD simulations revealed the complex interdomain interactions of cardiac tropin and revealed the calcium signaling mechanism (Genchev et al. 2021). Accordingly, Ca^2+^ binding, which constitutes the first signal of cardiac contraction, is characterized by three steps: (i) the capture of calcium, (ii) interactions of residues that transport Ca^2+^ ions to the binding site, and (iii) calcium-water exchange. The role of the switch peptide in this interaction is to ensure the hydrophobic pocket dynamics of the Ca^2+^-binding subunit Tn (TnC) N and to keep the hydrophobic pocket open or closed. These findings provide critical insights for the development of fine-tuned approaches to accessing the TnC N hydrophobic pocket for healthy cardiac contractile activity. The function of cardiac Tn associated with CAD is regulated by interactions between the N-terminal region of TnC and the C-terminal switch peptide of the troponin inhibitory subunit (TnI). To target the core binding site in the TnC NTD domain, a TnI-TnC interaction disrupting agent could be derived from the switch peptide (Xu et al. 2017). Recent reports have revealed that cardiac ion channels play a regulatory role in maintaining normal heart functions and pathophysiological processes of CAD, including its outcomes (Severino et al. 2020; Tripathi 2023). However, approaches to develop peptides that can regulate these ion channels are significantly limited due to the complex interactome of ion channels and the rich orchestration of these channels in cardiac cells (Papanikolaou et al. 2021; Marian et al. 2020). The complex interactomes that modulate ion channels, elucidated by current research, may pave the way for the in silico design of peptide therapeutics capable of facilitating healthy modulation of these channels against CAD in the near future.

### Inflammation

Inhibition of inflammatory pathways, one of the crucial mechanisms in the development of atherosclerosis, is one of the most promising methods for the development of therapeutic applications (Fig. 3). When the NF-κB nuclear localization sequence (NLS) is used to regulate the activation of NF-κB, a master regulator pathway of inflammation, it has been reported that it inhibits the importin α-mediated nuclear import of NF-κB and negatively regulates proinflammatory gene expression in vascular smooth muscle cells (Mallavia et al. 2013).

Aguas et al., subjected the binding site peptide sequence obtained from murine CCR2 to a series of homology models, energy calculations and Rosetta mutations to block chemokine C–C motif ligand 2 (CCL2) binding to the CCR2 receptor and proposed an 11 aa-long CCL2 blocking peptide (Aguas et al. 2023). Mimics of the kinase-inhibitory region of Suppressors of Cytokine Signaling 1 (KIR-SOCS1) have been proposed for many inflammation-related diseases, and the peptidomimetic PS5 has been reported to downregulate the expression of NADPH oxidase (NOX1 and NOX4) and proinflammatory genes, and to reduce plaque size, lipid content and monocyte/macrophage accumulation in vivo (Manna et al. 2020, 2021a, b). In ongoing studies, the same group has designed a chimeric peptide, KIRCONG, from the interface of the complex composed of the SOCS3, Janus kinase 2 (JAK2) and glycoprotein 130 (gp130) proteins. Moreover, they incorporated cyclic structures to improve the drug-like properties of SOCS3 and emphasized that its characterization under physiological conditions could lead to the development of potential therapeutics that could achieve SOCS3 bioactivity (Manna et al. 2021b). The regulation of inflammatory mechanisms by protein kinases, which are known to be essential in many crucial cellular functions, such as proliferation, cell cycle progression and proliferation, may provide novel perspectives for the development of potential therapeutic peptides against CAD (Qvit 2022).

Since inflammation is regulated by complex signaling pathways in the heterogeneous microenvironment of cardiac tissue, multiple pathways can be targeted to develop therapeutic options through anti-inflammatory mechanisms. One of these pathways, Pannexin1 (Panx1), allows the transport of some signaling molecules and metabolites between the cytoplasm and extracellular space (Narahari et al. 2021). In fact, the development of Panx1 inhibitors have a long history, but they suffer from low stability due to rapid hydrolysis of scissile amide bonds (Caufriez et al. 2023). To overcome the problems of the low proteolytic stability of Panx1 inhibitors developed in previous studies, the helical stapling method was applied. As a result, it was reported that the two analogs generated achieved a twofold increase in the inhibitory effect as well as a 30-fold increase in the stability profile under physiological conditions (Lamouroux et al. 2023).

This complex nature of inflammation has led to new perspectives for the treatment of CAD. Recent studies have suggested that the nucleotide-binding domain leucine-rich repeat and pyrin domain containing receptor 3 (NLRP3) inflammasome may be particularly critical for atherosclerosis (Kong et al. 2022; Soehnlein and Libby 2021; Tanase et al. 2023). Inhibition of NLRP3 inflammasome activation has been proposed for many inflammation-related diseases, and multiple approaches have been developed. For example, the potential inflammation regulatory roles of 9 peptides obtained as templates by structural analysis from distinct regions of inflammasome components for neurodegeneration were investigated, and as a result, it was shown that platforms for targeting these multiprotein inflammatory complexes could be designed with peptides (Sušjan et al. 2020). However, there is still a noticeable lack of therapeutic peptide applications targeting inflammasome complexes in CAD (Ye et al. 2023). In conclusion*, *in silico pipelines for designing peptides for use in the regulation of inflammatory pathways have provided promising strategies for the development of therapeutics for CAD. By targeting key regulators of inflammation, such as NF-κB, CCL2, and NLRP3 inflammasome, researchers have been able to design peptides that negatively regulate proinflammatory gene expression, block chemokine receptor binding, and inhibit inflammasome activation. However, a comprehensive examination of the downstream and systemic effects, as well as deep molecular analyses, are required to evaluate the effectiveness of inflammation-targeted approaches in cardiac tissue. Overall, the regulation of inflammatory mechanisms by peptide-based therapeutics has the potential to introduce promising new treatments for CAD in the near future.

### Regulation of lipoprotein levels

Since it was known from early studies that LDL-cholesterol increases the risk of atherosclerosis, the development of approaches to inhibit LDL accumulation is one of the most frequently focused mechanisms for the treatment of CAD (Fig. 3). The Gly^1127^-Cys^1140^ sequence in the CR9 domain of low-density lipoprotein receptor-related protein 1 (LRP1) is an important interaction domain for aggregated LDL in the atheric region (Costales et al. 2015). Peptides derived from this region have been shown to inhibit LDL aggregation by electrostatic interactions with domains critical for ApoB-100 conformational preservation (ApoB-100 conformation stabilization might guarantee the structural preservation of surface colesterol-enriched environments) (Benitez-Amaro et al. 2019). Furthermore, linear, cyclic, and alanine scanning derivatives of this sequence of 14 aa-long peptide were designed by combining structural- and ligand- based computational, molecular docking, and dynamics simulations and analyzed to generate a large repertoire of peptides that can be used for LDL aggregation (Benitez-Amaro et al. 2020).

Numerous signaling pathways and key proteins in these signaling pathways may contribute directly or indirectly to the development and progression of CVD. For example, the proprotein convertase subtilisin/kexin-type 9 (PCSK9) is a key mediator of cholesterol metabolism through protein–protein interactions with LDLR (Lin et al. 2018). In one of the early approaches, phage-displayed peptide libraries were screened, and a 13 aa-long linear peptide (named Pep2-8) was highlighted as a potential PCSK9 inhibitor (Zhang et al. 2014). Intensive efforts have been devoted to improving the design of more bioavailable peptides with high binding capacity by modifying this primary sequence. Modification approaches for Pep2-8 include 1-amino-4-phenylcyclohexane-1-carbonyl extension (~ 100-fold increase in affinity) (Burdick et al. 2020), conjugation with antagonistic peptides that can bind to the N-terminal groove of PCSK9 (20-fold increase in potency) (Zhang et al. 2017), and bioactive cyclization via various linkers (∼ 100-fold higher activity) (Tombling et al. 2021). In addition, Bourbiaux et al. reported novel analogs with 1000-fold better affinity than Pep2-8 using stapling as well as the addition of charged aa (Lys) to induce the helical conformation of Pep2-8 and thus protect it from enzymatic degradation (Bourbiaux et al. 2021).

For the development of PCSK9 inhibitors, Alleyne et al. used mRNA display screening and structure-based design techniques to design peptides that could inhibit the interaction between PCSK9 and LDLR (Alleyne et al. 2020). The researchers established a library derived from the starting form with serial modifications to obtain inhibitors with good permeability and oral bioavailability and emphasized potential cyclic PCSK9 inhibitors. These advances have impacted clinical practice, and initial findings of macrocyclic peptide-based approaches in CAD showed significant changes in LDL-C levels at all doses after 8 weeks of use (− 41.2%, − 55.7%, − 59.1%, and − 60.9% for 6 mg, 12 mg, 18 mg, and 30 mg, respectively), indicating that promising therapeutics may be able to be introduced to the market in the near future (Ballantyne et al. 2023; Kingwell 2023).

Another mediator lipoprotein associated with atherosclerosis is high-density lipoprotein (HDL). HDL exhibits numerous physiological effects that may play a role in atherosclerosis risk reduction. One approach to modulate HDL to exhibit atheroprotective properties is the design of sequences that could be apolipoprotein mimics (Fig. 3) (Leman et al. 2014). For example, the efficacy of Apolipoprotein A-I (ApoA-I) mimetic peptides in the efflux of HDL from the cell and consequently their therapeutic activity in atherosclerosis has been evaluated (Islam et al. 2020). By constructing a peptide library with distinct α-methylated Ala modifications to mimic the amphipathic helix structure of ApoA-I, researchers have clearly demonstrated that a stable α-helix is critical for cholesterol efflux. Komatsu et al., introduced a peptide sequence containing an artificial amphipathic helix that mimics the interaction site of Apolipoprotein C-II (apoC-II), which activates lipoprotein lipase (LPL) to reduce plasma triglycerides, and demonstrated increasing plasma clearance of TG-rich emulsions in vivo (Komatsu et al. 2019). However, structural modifications and with the assistance of MD simulations it was reported to improve the immunogenic properties of the apolipoprotein C-II mimetic peptide, as well as to enhance the ability of apoC-III to inhibit lipolysis and to design a new sequence (named D6PV) that provides a longer half-life (Wolska et al. 2020). In subsequent studies, researchers produced a short single helical mimic hydrocarbon stapled peptide similar to the last helix of ApoC2 (Sviridov et al. 2023). It was observed that this high stability peptide structure activated LPL and decreased plasma TG levels by 80–90% within 3 h in a mouse model of hypertriglyceridemia.

Another strategy to maintain HDL levels in cardiovascular tissue is to abrogate HDL interactions with human cholesteryl ester transfer protein (CETP), which contributes to disease by transferring cholesteryl ester from HDL to other lipoproteins. This protein functions by binding to its C-terminal amphipathic α-helix at its cognate site (termed the self-binding site). Zhu et al. halogenated the peptide derived from the C-terminal tail of CETP and reported an almost threefold increase in affinity over the native helical conformation (Zhu et al. 2016). Moreover, when a hydrocarbon-stapled/halogenated helical peptide was designed, molecular dynamics simulations have shown that binding affinities of more than fivefold can be achieved (Zhu et al. 2020). Researchers have successfully developed peptides that inhibit LDL aggregation and lower LDL-C levels by targeting key mediators of cholesterol metabolism, such as LDL-cholesterol and PCSK9.Utilizing computational simulations and structural modifications has resulted in the creation of peptides with enhanced bioavailability and strong binding capacity, leading to significant reductions in LDL-C levels observed in clinical trials. Moreover, the design of sequences resembling apolipoprotein A-I and C-II has demonstrated potential in modulating HDL to manifest atheroprotective properties.

It is apparent that strategies aimed at regulating lipoprotein metabolism bear notable potential in the development of therapeutic peptides for CAD. However, regardless of the capabilities of in silico methods, given the intricate regulation of this metabolism across various signaling pathways, comprehensive exploration is imperative to elucidate the effects of these promising therapeutics.

## Conclusion and future perspective

The safety concerns arising from the prolonged and combined use of conventional small molecule-based therapeutic agents have led to a growing interest in biological molecule-based approaches for the treatment of CAD, particularly the use of peptides, which exhibit superior properties such as high target specificity, low accumulation in tissues and low toxicity. Primary findings with peptides derived from other natural sources or protein–protein interfaces have demonstrated the potential of peptide-based therapeutic approaches. Today, tools and software developed through state-of-the-art approaches have introduced a new era in therapeutic peptide design.

In silico methods have become prominent in the design of peptides that display activity by interacting with specific targets in CAD. For example, it is an undisputed fact that virtual screening methods to determine the most appropriate peptide sequence have led to new insights for the development of CAD-specific therapeutic peptide platforms (Mahmoodi-Reihani et al. 2020). Solving protein‒peptide interactions using molecular docking or dynamics simulations has led to significant insights for wet-laboratory applications. However, implementing the workflows of these methods requires expertise in the biochemistry of peptides and pathophysiology of the disease as well as specialized knowledge in the establishment of infrastructure using high-performance computing methods. Here, the most pragmatic approaches may be to implement ML methods for specific targets in CAD and launch them as user-friendly servers. However, in order to make biological activity predictions for CAD, studies are needed to build comprehensive and sufficient datasets. At the same time, there is still a need for new perspectives for the utilization of advanced technology developments in structural biology, such as AlphaFold, in the development of CAD-specific ML applications. Considering all these, it is clear that in silico methods, which have great potential in designing safe therapeutic peptides for CAD, have made significant progress. However, especially the integration of conventional structural biology tools with ML applications may lead to technologies that are currently far beyond current in the development of therapeutic peptides.

In addition, peptide sequences designed solely by in silico methods may require tailored modifications. For example, peptide sequences composed of natural aa’s exhibit poor drug-like properties (absorption, low stability to proteolytic digestion, and fast clearance) under physiological conditions. Therefore, novel strategies for the design of therapeutics with high bioavailability can be achieved by evaluating a wide range of peptide chemical modifications as well as in silico methods (Li et al. 2020; Moiola et al. 2019). On the other hand, in addition to therapeutic purposes alone, strategies can be developed for peptides to facilitate the targeting of therapeutics to specific tissues through multidisciplinary approaches (Mentkowski and Lang 2019).

In a holistic context, it is noteworthy that although today there are tools with great potential for in silico peptide design, this potential is underutilized for the development of therapeutic applications in CAD. A much more comprehensive and cutting-edge approach is needed to transform all these strategies for therapeutic peptide design into promising treatments for millions of CAD patients by preventing or treating the development of CAD-related complications.

## Data Availability

Not applicable.
